# A biological question and a balanced (orthogonal) design: the ingredients to efficiently analyze two-color microarrays with Confirmatory Factor Analysis

**DOI:** 10.1186/1471-2164-7-232

**Published:** 2006-09-12

**Authors:** Anne PG Crijns, Frans Gerbens, A Edo D Plantinga, Gert Jan Meersma, Steven de Jong, Robert MW Hofstra, Elisabeth GE de Vries, Ate GJ van der Zee, Geertruida H de Bock, Gerard J te Meerman

**Affiliations:** 1 Department of Gynecologic Oncology, University Medical Center Groningen and University of Groningen, PO-box 30.001, 9700 RB, Groningen, The Netherlands; 2 Department of Medical Genetics, University Medical Center Groningen and University of Groningen, PO-box 30.001, 9700 RB, Groningen, The Netherlands; 3 Department of Medical Oncology, University Medical Center Groningen and University of Groningen, PO-box 30.001, 9700 RB, Groningen, The Netherlands; 4 Department of Epidemiology and Statistics, University Medical Center Groningen and University of Groningen, PO-box 30.001, 9700 RB, Groningen, The Netherlands

## Abstract

**Background:**

Factor analysis (FA) has been widely applied in microarray studies as a data-reduction-tool without any a-priori assumption regarding associations between observed data and latent structure (Exploratory Factor Analysis).

A disadvantage is that the representation of data in a reduced set of dimensions can be difficult to interpret, as biological contrasts do not necessarily coincide with single dimensions. However, FA can also be applied as an instrument to confirm what is expected on the basis of pre-established hypotheses (Confirmatory Factor Analysis, CFA). We show that with a hypothesis incorporated in a balanced (orthogonal) design, including 'SelfSelf' hybridizations, dye swaps and independent replications, FA can be used to identify the latent factors underlying the correlation structure among the observed two-color microarray data. An orthogonal design will reflect the principal components associated with each experimental factor. We applied CFA to a microarray study performed to investigate cisplatin resistance in four ovarian cancer cell lines, which only differ in their degree of cisplatin resistance.

**Results:**

Two latent factors, coinciding with principal components, representing the differences in cisplatin resistance between the four ovarian cancer cell lines were easily identified. From these two factors 315 genes associated with cisplatin resistance were selected, 199 genes from the first factor (False Discovery Rate (FDR): 19%) and 152 (FDR: 24%) from the second factor, while both gene sets shared 36. The differential expression of 16 genes was validated with reverse transcription-polymerase chain reaction.

**Conclusion:**

Our results show that FA is an efficient method to analyze two-color microarray data provided that there is a pre-defined hypothesis reflected in an orthogonal design.

## Background

DNA microarrays are often used to identify genes that are differentially expressed among different predefined classes of samples. In a two-color microarray system both RNA samples are separately labeled with different colors, mixed, and hybridized together to an array. The ratio of the two-color signal intensities for each spot represents a relative measure of gene expression. There are different types of design of two-color microarrays for identifying differentially expressed genes, such as the reference design (most commonly used), balanced block design, and loop design [1].

Two-color microarray data analysis generally consists of two stages. In the first stage, microarray data are filtered and normalized, e.g. adjusted for some of the systematic and technical variation that affects the measured gene expression levels. There are different methods to correct (normalize) microarray data for systematic and technical variation [2-8]. In the second stage of microarray data analysis, statistical methods are used to identify the genes that are differentially expressed between the different classes of samples. Most of these statistical methods use similar basic statistics and differ mainly in their determination of the significance threshold. Therefore, when applied to microarray data they give very similar overall results [9,10].

Factor analysis (FA) can be applied as a data-reduction-tool without any a-priori assumption regarding associations between observed data and latent structure (Exploratory Factor Analysis, EFA). For this purpose FA has been widely applied in microarray studies [3]. A disadvantage of EFA is that the representation of data in a reduced set of dimensions can be difficult to interpret. On forehand the interpretation of the extracted factors is not fixed and biological contrasts do not necessarily coincide with single dimensions.

Yet, FA could be very well used for gene selection when it is applied as an instrument to confirm what is expected on the basis of pre-established hypothesis (Confirmatory Factor Analysis, CFA) [11]. When two-color microarray experiments are designed such that a hypothesis can be defined a-priori regarding the latent structure among the observed two-color microarray data, biologically relevant factors can be easily identified from which genes can be selected (as the correlation structures of the biologically relevant factors with the arrays should mirror the applied design).

In this paper we will illustrate CFA as a powerful statistical tool to analyze DNA microarray data. As a model a microarray study is used in which the differences in gene expression related to cisplatin resistance are measured, using two-color microarrays, for four ovarian cancer cell lines (A2780, CP70, C30 and C200), which only differ in their degree of cisplatin resistance. A2780, the parental cell line, is cisplatin sensitive and its sublines CP70, C30 and C200 are increasingly resistant to cisplatin (5, 75 and 125 times compared to A2780, respectively).

### Background methodology

#### Confirmatory Factor Analysis

The fundamental idea underlying the factor analytic models is that not all causative variables can be directly observed. These unobserved variables are referred to as latent structure of factors. Information about factors can be obtained by inspecting how the factor elements are formed from linear combinations of the observed variables. In an EFA, there is no specified structure of the relationships among the variables under study. In a confirmatory factor analysis the retrieved factors should reflect contrasts that correspond to differences in sample characteristics. There are a-priori defined constraints on the relationships among the latent factors and the variables under study. It is in this sense that the FA is thought of as confirmatory.

**Figure 1:**
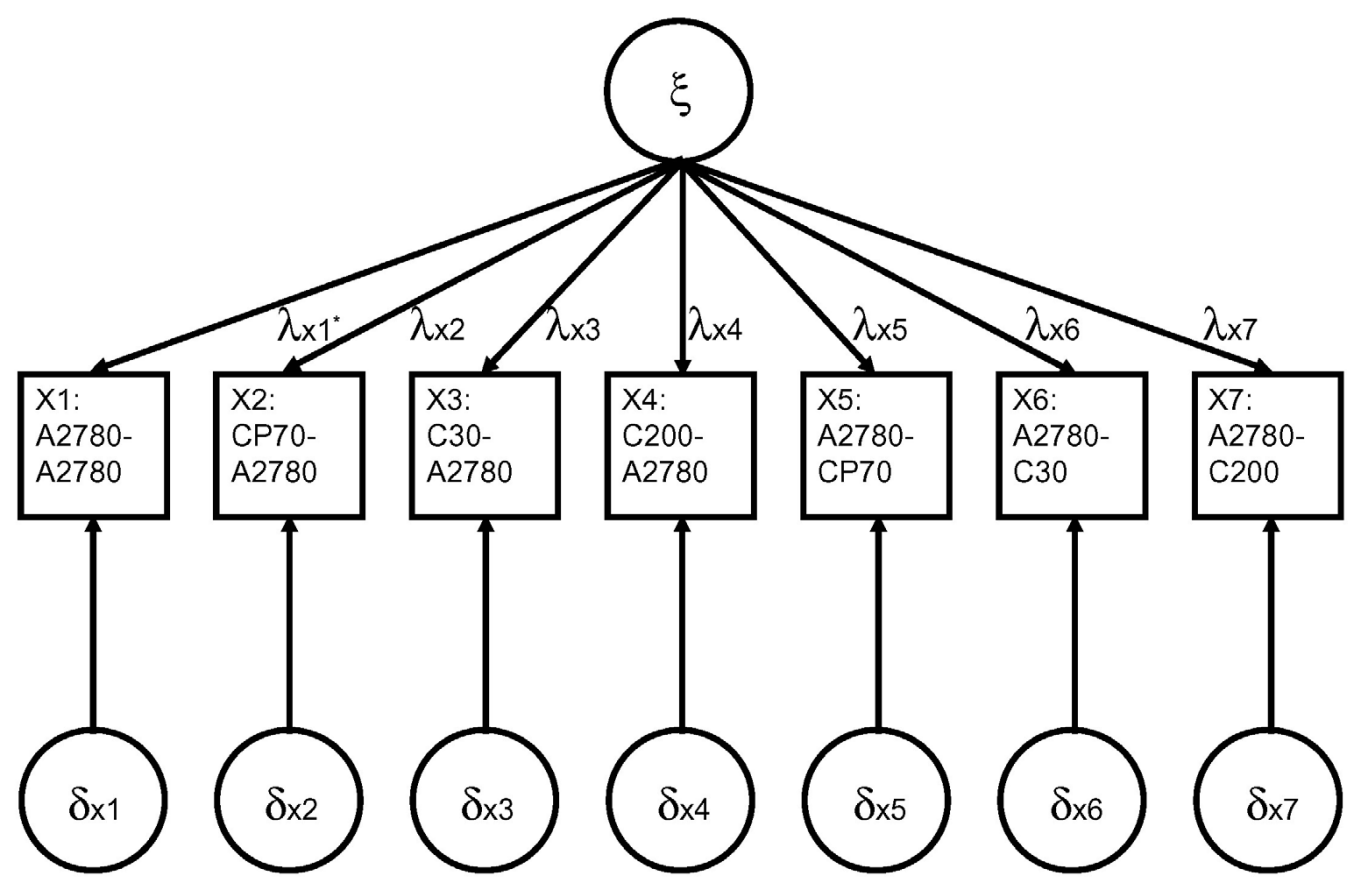
**Confirmatory Factor Analysis Model of the microarray study**. The differences in gene expression related to cisplatin resistance are measured, using two-color microarrays, for four ovarian cancer cell lines (A2780, CP70, C30 and C200), which only differ in their degree of cisplatin resistance. The latent factors representing level of cisplatin resistance are depicted as a circle at the top of the figure. The squares represent the observed variables, e.g. the microarrays. The arrows connecting the latent factor and the arrays illustrate the loadings of the arrays on the latent factor. (In our model the latent factors do not load on the 'SelfSelf' hybridization, X1 (λ_x1_^* ^= 0)). The circles at the bottom of the picture symbolize the measurement errors. (This design was performed in triplicate with three independent cultures of the ovarian cancer cell lines).

In the here illustrated model, differences in gene expression between four ovarian cancer cell lines (A2780, CP70, C30 and C200) are related to their degree of the cisplatin resistance. The level of cisplatin resistance can be considered as the latent factor among the observed gene expression data. The latent factors, referred to as ξ, are depicted as a circle at the top of Figure 1. The ξ is imperfectly measured by a number of observed variables, e.g. two-color microarrays. While it is assumed that the number of observed variables in X is greater than the number of latent factors in ξ, there is no a-priori assumption about the exact number of latent factors. In our example, seven two-color microarrays are used, referred to as X1 to X7, and they are indicated by the squares in Figure 1. The measurement errors in the observed variables, referred to as δ_X1 _to δ_x7_, are depicted as circles at the bottom of the Figure 1. X1 to X7 are said to be effected by or load on ξ, the level of cisplatin resistance. The loadings, referred to as λ_X1 _to λ_X7_, are indicated by the arrows connecting the latent factor to the observed variables.

In a microarray experiment there are *i *= 1,..., n performed arrays and *j *= 1,..., p probed genes. Typically, *j *is in the order of thousands, while *i *is in the order of 10–100. The gene expression measurements of the microarray experiment are represented by a matrix X = [x_*1*_,...,x_*n*_] of dimension *n *× *p*, with arrays as columns and genes as rows. Each element x_*ij *_corresponds to the gene expression measurement for the *j*th gene of the *i*th array. The expression level x_*ij *_of each gene can be reconstructed by the standard linear equation

xij=∑s=1Sλsiξsj+δsi
 MathType@MTEF@5@5@+=feaafiart1ev1aaatCvAUfKttLearuWrP9MDH5MBPbIqV92AaeXatLxBI9gBaebbnrfifHhDYfgasaacH8akY=wiFfYdH8Gipec8Eeeu0xXdbba9frFj0=OqFfea0dXdd9vqai=hGuQ8kuc9pgc9s8qqaq=dirpe0xb9q8qiLsFr0=vr0=vr0dc8meaabaqaciaacaGaaeqabaqabeGadaaakeaacqqG4baEdaWgaaWcbaGaemyAaKMaemOAaOgabeaakiabg2da9maaqahabaGaeq4UdW2aaSbaaSqaaiabdohaZjabdMgaPbqabaGccqaH+oaEdaWgaaWcbaGaem4CamNaemOAaOgabeaaaeaacqWGZbWCcqGH9aqpcqaIXaqmaeaacqWGtbWua0GaeyyeIuoakiabgUcaRiabes7aKnaaBaaaleaacqWGZbWCcqWGPbqAaeqaaaaa@47D8@

This means that the observed expression for the *j*th gene of the *i*th array is the sum of its activities in each of *s *latent factors (contrasts in the levels of cisplatin resistance between the 4 cell lines), denoted by ξ_*sj*_, weighted by the activity of this latent factor in array *i*, denoted by λ_*si*_, plus some array-specific noise δ_*si*_.

This can be represented in matrix format as:

X = Λξ + δ

Where Λ is the *p *× *p *matrix of factor loadings (the correlation structure of each of the latent factors *s *with the arrays *i*) and ξ is the *n *× *p *matrix of factor scores (the levels of activity of each gene *j *within each of the *s *latent factors) and δ is the matrix of residuals as result of dimension reduction.

By subtracting the mean from both the observed and latent variables it is possible to define the covariance matrix of a vector of variables in terms of expectations of vector products. In addition, it is assumed that the latent factors are uncorrelated (i.e. orthogonal). For that, in the here-illustrated example, we applied the method of Singular Value Decomposition (SVD), the equivalent of principal components analysis. This method assumes that the extracted factors are uncorrelated and orders the factors according to percentage explained variation (successive factors account for less and less variation overall). The number of extracted factors can maximally be equal to the total number of arrays.

The confirmatory factor model is identified if the constraints have been imposed in such a way that there is a unique set of parameters that can generate the covariance structure. More specific, if a parameter can be solved for in terms of the variances and covariances of the observed variable, it is identified. The constraint that retrieved factors have to be orthogonal is applied as some patterns underlying the microarray data are expected to be correlated with biological processes and others with experimental artifacts.

After identification has been established, estimation can start. The objective in estimating the factor model is to find estimates of the latent factors and errors that reproduce the sample matrix of covariances as closely as possible. The fitting function used in the here-illustrated example, is the Unweighted linear Least Squares (ULS). The problem of scale dependency was solved by performing the analyses on the correlation structure instead of the covariance structure.

### Hybridization design

The hybridization design of the micro-arrays was as follows:

- The design was balanced: the four ovarian cancer cell lines were hybridized to the microarrays according to a reference design, indicating that all samples were hybridized against the cisplatin sensitive cell line A2780 (common reference). The microarray data were expressed as Cy5/Cy3 ratios for each spot.

- The design included a 'SelfSelf' hybridization, X1 in Figure 1. Note that all observed microarray data were expected to load on the latent factor ξ, except for X1 (λ_x1 _= 0). This is because there should be no biological difference between the two-color signal intensities of 'SelfSelf' experiments and therefore the 'SelfSelf' hybridization was assumed not to load on the latent factor.

- The design included dye swaps, X2 and X5, X3 and X6, X4 and X7 in Figure 1. CP70, C30 and C200 were labeled with Cy5 and hybridized against Cy3-labeled A2780, X2, X3, and X4 in Figure 1, respectively. Then the dyes were swapped: CP70, C30 and C200 were labeled with Cy3 and hybridized against Cy5-labeled A2780, X5, X6, and X7, respectively. The sign of the loadings of X2, X3 and X4 were assumed to be opposite to the signs of the loadings of X5, X6 and X7, as these were the dye swaps. The magnitude of the loadings of X2 and X5, X3 and X6, X4 and X7 were assumed to be similar, as the same ovarian cancer cell lines were hybridized to these arrays. Once the latent factors had been identified using their correlation structure with the observed variables, differences in level of cisplatin resistance would appear as contrasts between similar arrays after correction (sign change) for dye swaps.

- The design was performed in triplicate with three independent cultures of the cell lines. It was expected that replicate arrays, e.g. the two replicates of X1 to X7, would show the same loadings.

### Analysis

First the observed variables were standardized, i.e. FA was separately applied to the Cy5 and Cy3 microarray data to subtract the variation all arrays had in common. The first factor explaining the largest part of the variation, could be considered as variation the arrays have in common [3]. This factor could be used for array quality control, as it would have lower or distinctly different correlations with arrays of lesser quality. In addition, plotting the standardized Cy5 signal intensities against the standardized Cy3 signal intensities, allowed us to test whether the hybridizations were non-competitive [12]. However, from a mathematical point of view there is no objection to directly subjecting Cy5/Cy3 ratios to FA.

In the second step, CFA was performed to uncover which of the factors coincided with differences in levels of cisplatin resistance between the four ovarian cancer cell lines and to select the genes with the highest loadings on those factors.

It was assumed that a random process leading to non-normal distributions would likely affect all extracted factors (biological and non-biological) to an equal degree. Therefore, it was assumed that the statistical distribution of the gene expression data under the null hypothesis could be estimated from the factors that most likely represent noise. Because we expected that the biological factors would result in genes with more extreme scores than those present in the non-biological factors we performed the analysis on scores with equal rank. First, the elements of each retained (biological) factor were rank-ordered and normalized to a mean of zero and to a standard deviation of one. Then the elements with the same rank for each of the factors representing noise were averaged and renormalized. Because the distribution affected by biological effects has more weight in the tails of the distribution, the elements with the most extreme scores will be larger in absolute value than the elements with the same rank from the rank averaged factors. Subsequently, the genes with the same rank with the largest difference values (≤ -1 and ≥ 1) from the averaged distribution were selected. This happened to result in selecting the genes with the most extreme scores. The threshold below or above (≤ -1 and ≥ 1) genes were selected was used to obtain a false discovery rate (FDR) by observing how many elements of the rank averaged factors were below or above this threshold.

For a script of the applied method, the corresponding author can be contacted.

## Results

### First step of the FA procedure: standardization of the Cy5 and Cy3 data

**Figure 2:**
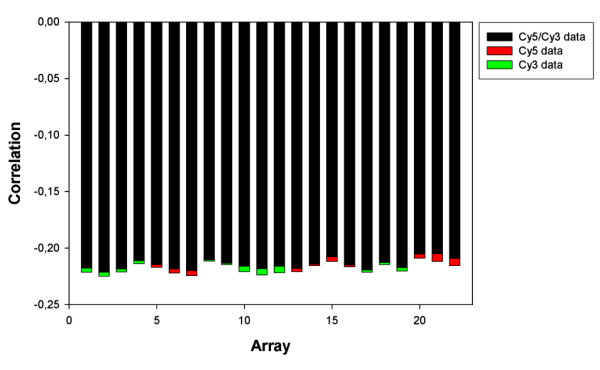
Correlations between the Cy5 and Cy3 data from each array, respectively, and the first factor retained with FA.

The first factors from the Cy5 and Cy3 data explained 85% of the total variation and represented variation common to all arrays. Figure 2 shows that the correlations between the Cy5 and Cy3 data of each array and the first factor were highly similar. This figure also indicates that the quality of the arrays was very comparable. By subtracting this common variation from the Cy5 and Cy3 data all gene specific variation that does not contribute to differences between arrays was eliminated (i.e. the Cy5 and Cy3 data were standardized by subtracting the first factor).

**Figure 3:**
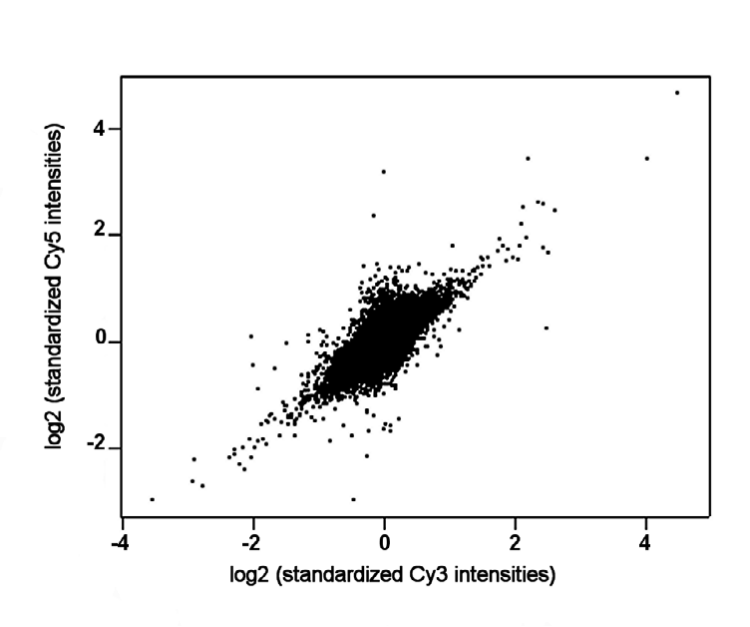
Scatterplot of the standardized Cy5 signal intensities against the standardized Cy3 signal intensities of all arrays.

After elimination of the common variation, the standardized Cy5 and Cy3 signal intensities of all arrays combined showed a positive correlation (Figure 3). When Cy5 signal intensities in a specific array were lower than average, also Cy3 signal intensities were lower than average, and reversely, implying that the hybridizations were non-competitive. The phenomenon of non-competitive hybridization as seen in our cDNA microarrays was recently also described for long-oligonucleotide microarrays by 't Hoen and colleagues [12].

### Second step of the FA procedure: identification of the biologically relevant factors

**Figure 4:**
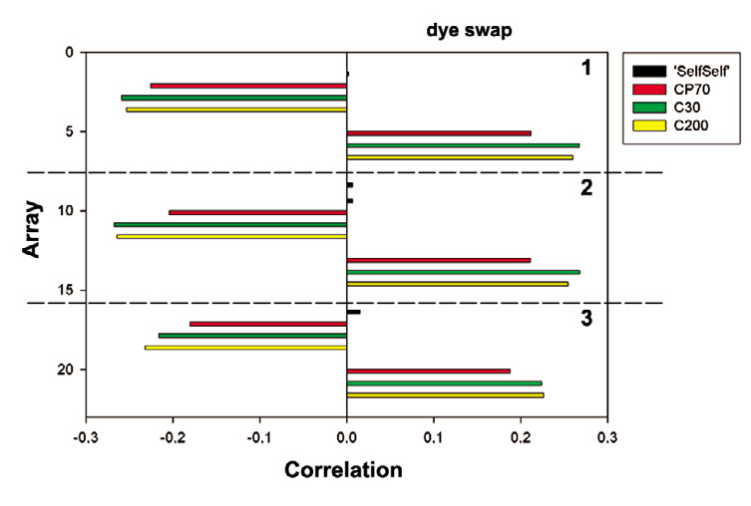
Correlations between the standardized Cy5/standardized Cy3 ratios from each array and the first factor retained with FA.

**Figure 5:**
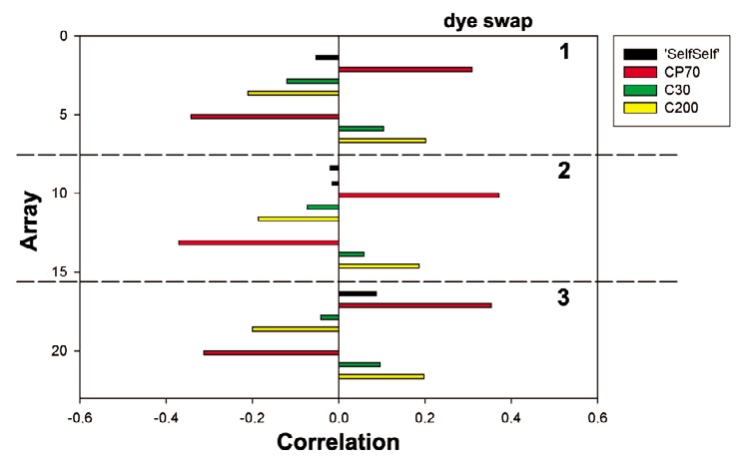
**Correlations between the standardized Cy5/standardized Cy3 ratios from each array and the second factor retained with FA.**

In the second step, the factors representing differences in cisplatin resistance between the four ovarian cancer cell lines were identified. We identified two factors (the first and second factor) of which the correlation structures with the observed variables, e.g. arrays, reflected the balanced reference design (Figures 4 and 5).

The first factor explained a considerable part of the remaining variation between arrays (40%). The first factor did not correlate with the 'SelfSelf' hybridizations. Furthermore, the sign of the loading of dye swap experiments on the first factor was opposite and replicate experiments showed the same loading (Figure 4). After the sign of the dye swaps was changed, all the arrays showed similar loadings on the first factor. Thus, this factor reflected the contrast between A2780 (used as reference in each array) and CP70 + C30 + C200. The microarray data of the more resistant ovarian cancer cell lines C30 and C200 had a consistently higher (about 4%) loading on this factor than the data of the less resistant cell line CP70.

The second factor was only associated with a minor amount of variance (11%), but its correlation structure with the arrays reflected 'SelfSelf' hybridizations, dye swaps and replicate experiments. After correction for dye swaps, this factor could be interpreted as a contrast between CP70 and C30 + C200 (Figure 5), as the sign of the loading of the microarray data of CP70 on the second factor was opposite to the sign of the loadings of the data of C30 and C200. The absolute weight of the microarray data of C30 (mean: 8%) was much lower than that of the data of C200 (mean: 19%) and CP70 (mean: 25%), so this factor reflected mainly the difference between the least and most cisplatin resistant cell line.

After the factors coinciding with biological contrasts (differences in cisplatin resistance between the cell lines) had been identified, the genes responsible for these contrasts were selected as the most extreme ones from the first and second factor. From the first factor 199 genes (FDR: 19%) were selected and from the second factor 152 genes (FDR: 24%). Both gene sets had 36 genes in common.

**Figure 6:**
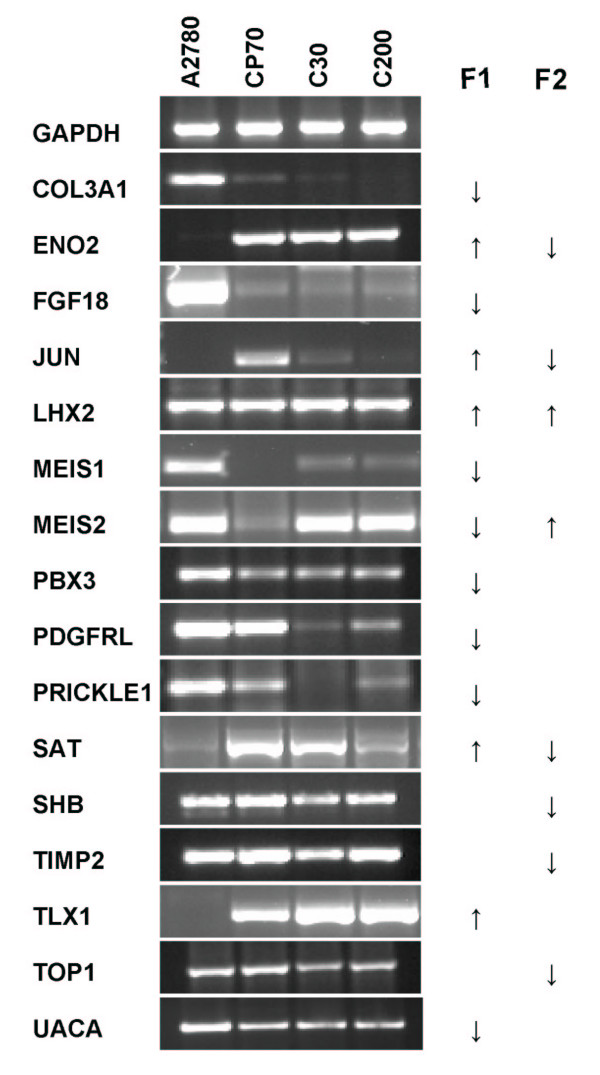
**RT-PCR results for 16 genes of the 4 ovarian cancer cell lines**. F1, up- (↑) or down- (↓) regulated in CP70 + C30 + C200 compared to A2780. F2, up- (↑) or down- (↓) regulated in C30 + C200 compared to CP70.

Of the 199 genes selected from the first factor, the expression of 99 genes was up-regulated and the expression of 100 genes was down-regulated in CP70 + C30 + C200 compared to A2780. Of the 152 genes selected from the second factor, the expression of 24 genes was up-regulated and the expression of 128 genes was down-regulated in C30 + C200 compared to CP70. To validate the expression of genes selected from the biological factors, reverse transcription-polymerase chain reaction (RT-PCR) was performed for 16 genes with GAPDH as a control: COL3A1, ENO2, FGF18, JUN, LHX2, MEIS1, MEIS2, PBX3, PDGFRL, PRICKLE1, SAT, SHB, TIMP2, TLX1, TOP1 and UACA. Figure 6 demonstrates that the differential expression pattern of the 16 genes, as determined with RT-PCR, was comparable to the FA results of the microarray data, confirming the reliability of our analysis of the microarray data. Additionally, in Table 2 is shown that there is overlap between our gene lists and gene lists from other groups who have profiled A2780 and its cisplatin/oxaliplatin resistant subline(s), confirming our results [13-16]. Furthermore, FatiGO was used to annotate the genes with Gene Ontology (GO) terms (biological process and molecular function) and to compare the distribution of the main GO terms between the gene list selected from the first and second factor [17]. As shown in Table 3, the distributions of the main GO terms were not significantly different between the two groups of genes.

## Discussion

In general FA, as effected by SVD, is applied to two-color microarray data for summarizing, filtering and pre-processing data (EFA), although several studies have shown FA can be used for gene selection [18-29]. A weakness of straightforward application of FA to microarray data without any a-priori expectations regarding the latent structure among the observed data is that there is no straightforward way of objectively assessing model performance. However, in our microarray study we have shown that rather than applying FA directly to the analysis of microarray data, when the structure of the relationships among the variables (e.g. arrays) is specified a-priori, reflected by the design of the study, FA is an efficient method to analyze two-color microarray data.

Based on this pre-defined hypothesis two latent factors coinciding with differences in cisplatin resistance between four ovarian cancer cell lines were easily identified. The first factor retrieved during the fist step of FA represented the common variation of arrays and the first two factors retrieved during the second step represented differences between arrays. The variation of the arrays is generally explained by only a small number of factors, of which the first (the major source of variation) represents variation the arrays have in common [18,20-24,26,27]. One of the two factors that represented differences between arrays was interpreted as the contrast between the cisplatin sensitive A2780 cells and the cisplatin resistant CP70, C30 and C200 cells. The other factor was explained as the contrast between the mild cisplatin resistant CP70 cells and the extreme cisplatin resistant C30 and C200 cells. From the first factor 199 genes and from the second factor 152 genes were selected and 36 genes were shared by both gene sets. This overlap makes it very plausible that the two retrieved factors are indeed biologically meaningful. It is biologically plausible that genes that are important for the difference between cisplatin sensitive cells and cisplatin resistant cells are also responsible for the difference in the degree of cisplatin resistance.

By using SVD for the computation of the latent factors underlying the microarray data, we obtain uncorrelated (i.e. orthogonal) factors. Therefore the outlier genes selected from each factor are not necessarily the same. The expected number of outlier genes common to both factors under the hypothesis of no relation is 1.6, which is much lower than the actually found number of 36. Comparison of selected genes sets from biologically relevant factors between arrays may, thus, be an important tool to validate that the factors are indeed biologically meaningful. The biological relevancy of the factors was also supported by the finding that the loadings of the expression data of the ovarian cancer cell lines on the two contrasts differed which could also be attributed to the differences in the levels of cisplatin resistance of the cell lines. Furthermore, the FA data were supported by RT-PCR results for 16 genes and literature [13-16].

Analyzing microarray data with CFA has several advantages. With only one algorithm, that is available in any standard statistical software package, both stages of microarray data analysis can be performed. By applying FA, variation in microarray data caused by biological differences can be separated from variation related to the microarray technique. By using SVD, we assumed that some latent factors are expected to be correlated with biological processes and others with experimental artifacts. So, applying FA to microarray data also means that to select differentially expressed genes between different classes of samples, the classes do not have to be defined a-priori.

An advantage of using ULS as fitting method is that no assumption about the distribution of the microarray data has to be made. Other microarray analysis methods often assume that the gene expression data follow normal distribution, but in reality the distribution is not necessarily symmetric and its tails can differ in size and shape. A random process leading to non-normal distributions will likely affect all extracted factors (biological and non-biological) to an equal degree. Therefore, the statistical distribution of the gene expression data can be estimated from the factors that most likely represent noise.

The problem of scale dependency was solved by performing the analyses on the correlation structure instead of the covariance structure. In this analysis we were not hindered by the difficulty that for ULS no formal tests are available. Instead the false discovery rates for genes selected from biological factors were calculated as indicators for their quality. In addition, there was no real need to assess the fit of the model as the retrieved factors reflected the design of the study, and the false discovery rate was calculated being an indicator for the quality of our proposed cisplatin resistant genes.

The identification of biologically meaningful factors is uniquely dependent on the data and cannot be guaranteed. By randomization and balancing of possible confounders of microarray experiments, such as the order of processing (during one of the many steps of microarray experiments), the systematic (biological and instrumental) effects will be orthogonal and are likely to show up as factors. Rotation of the Factor analysis structure is eventually possible and will not result in a substantial decrease in the amount of variance explained.

Another disadvantage may be that to use CFA the design of the two-color microarray study ideally should include 'SelfSelf' hybridizations, dye swaps and independent replications, which may not always be the most efficient design due to the increasing number of arrays. Biological factors are not easily identified when FA is applied to designs not including orthogonal contrasts, i.e. without 'SelfSelf' hybridizations or dye swaps, necessary to pre-define the structure of the relationships among the variables (e.g. arrays). Examples of such designs not including orthogonal contrast are balanced block designs or loop designs [30,31].

## Conclusion

In conclusion, our results show that FA is an efficient method to analyze two-color microarray data provided that there is a pre-defined hypothesis reflected in an orthogonal design.

## Methods

### Sample preparation and microarray experiments

Total RNA was isolated from the ovarian cancer cell lines A2780, CP70, C30 and C200 (kindly provided by T.C. Hamilton, Fox Chase Cancer Centre, Philadelphia, US) in three independent experiments by guanidine isothiocyanate treatment and subsequent purification by cesium chloride ultracentrifugation. After DNAse treatment, the RNA was linearly amplified according to the T7 amplification protocol of the Central Microarray Facility of The Netherlands Cancer Institute [32]. Each amplified RNA (cRNA) sample was then independently labeled with Cy3 (green) and Cy5 (red).

The labeled samples were hybridized to the 18K cDNA microarrays produced at the Central Microarray Facility of the Netherlands Cancer Institute (NCI, Amsterdam, The Netherlands) according to the balanced reference design described in Table 1 and Figure 1; All four ovarian cancer cell lines, A2780, CP70, C30 and C200 were hybridized against A2780 (common reference). Dye swaps were performed for all experiments, except for the 'SelfSelf' hybridization of A2780. This design was used for the 3 independent RNA isolations from the cell lines (3 independent cell cultures). In addition one extra 'SelfSelf' was performed in the second replication of the design, resulting in a total of 22 hybridizations [32].

Fluorescent images of the microarray slides were obtained with the Affymetrix GMS428 scanner (Santa Clara, CA). For both fluorophores, signal intensities for each spot were quantified by dedicated IMAGENE 5.6 software (Biodiscovery, Marina Del Rey, CA).

### Reverse transcription-polymerase chain reaction (RT-PCR)

Sixteen of the differentially expressed genes were further assessed in the 4 ovarian cancer cell lines by RT-PCR. cDNA was synthesized from 5 μg total RNA using oligo dT primers and MMLV transcriptase. The primer sequences and PCR conditions for the genes are described in Table 4. PCR products were electrophorized in a 1.2% agarose gel in 1x Tris-borate EDTA buffer.

### Gene Ontology (GO) annotation

FatiGO was used to annotate the genes with GO terms (biological process and molecular function) and to compare the distribution of the main GO terms between the gene list selected from the first and second factor [17].

## Abbreviations

FA: Factor Analysis

EFA: Exploratory Factor Analysis

CFA: Confirmatory Factor Analysis

FDR: False Discovery Rate

SVD: Singular Value Decomposition

ULS: Unweighted linear Least Squares

RT-PCR: reverse transcription-polymerase chain reaction

GO: Gene Ontology

## Authors' contributions

APGC performed the microarray experiments, applied FA and BRB analysis and wrote the manuscript, FG supervised BRB analysis and helped writing the manuscript, AEDP assisted in applying FA, GJM performed the microarray experiments, GHDB assisted in writing the manuscript, GJTM designed the microarray study, implemented FA, performed the statistical analyses and participated in writing the manuscript. SDJ, RMWH, EGEDV, and AGJVDZ designed the study and revised the manuscript critically for important intellectual content. All authors read and approved the final manuscript.

## Figures and Tables

**Table 1:** Overview of the balanced reference design*

*Hybridization design*	*Array*	*Cy3 (green)*	*Cy5 (red)*
SelfSelf	1	A2780	A2780
Ref-CP70	2	A2780	CP70
Ref-C30	3	A2780	C30
Ref-C200	4	A2780	C200
CP70-Ref	5	CP70	A2780
C30-Ref	6	C30	A2780
C200-Ref	7	C200	A2780

* This design was used for the 3 independent RNA isolations from the ovarian cancer cell lines A2780, CP70, C30 and C200 (3 independent cell cultures). In addition one extra 'SelfSelf' was performed in the second replication of the design, resulting in a total of 22 hybridizations.

**Table 2:** Comparison of our gene list with gene lists described in literature

*Reference: Cell lines*	*A2780 vs. CP70 + C30 + C200*		*CP70 vs. C30 + C200*
	
	*Up-regulated*	*Down-regulated*	Down-regulated*
[13]: 6 cisplatin resistant cell pairs, including A2780 and CP70	JUN (↑) and IFITM1 (↓)	MRC2(↓)	
[14]: A2780 and oxaliplatin resistant C10B	FER1L3 (↑), LIPA (↑), IFITM1 (↑), NMI (↑) and ALCAM (↑)	SOC2 (↓), MID1(↓), TFPI (↓), MMRN (↓), CCR1 (↓) and NID2 (↑)	MMP3 (↑), SPARC (↑), FER1L3 (↑), TM4SF1 (↓), CRIM1 (↓) and PEG10 (↓)
[16]: 4 oxaliplatin resistant cell pairs, including A2780 and R4	NFE2L1 (↑), IFITM1 (↓)	TIP120B (↑)	TIMP2 (↑), COTL1 (↑), IL1R1 (↑), SPARC (↑) and SLC4A2 (↓)
[15]: A2780, cisplatin resistant ACR6 and ACRP		S100A11 (↓) and SLC25A6 (↓)	PEG10 (↑), TMSB4X (↓) and COL5A2 (↓)

The arrows behind the genes indicate whether the gene was up-regulated (↑) or down-regulated (↓) in the cisplatin/oxaliplatin resistant subline(s) compared to the cisplatin/oxaliplatin sensitive parental cell line according to microarray or SAGE data of other groups. * The genes selected from factor 2 that were also described in literature were all down-regulated in C30 + C200 compared to CP70.

**Table 3:** Comparison of the main Gene Ontology categories among the gene lists

*Gene Ontology: Level 3*	*A2780 vs. CP70 + C30 + C200*	*CP70 vs. C30 + C200*	
		
	*N° Genes (%)^1^*	*N° Genes (%)^2^*	*P*
*Biological Process*			
Cellular physiological process	60 (81)	39 (78)	0.82
Metabolism	43 (58)	29 (58)	1
Regulation of cellular process	24 (32)	15 (30)	0.85
Organismal physiological process	23 (31)	10 (20)	0.22
Regulation of physiological process	22 (30)	16 (32)	0.84
Cell communication	20 (27)	13 (26)	1
Localization	18 (24)	10 (20)	0.66
Response to stress	13 (18)	5 (10)	0.30
Response to biotic stimulus	10 (14)	5 (10)	0.78
Cell adhesion	10 (14)	3 (6)	0.36
Negative regulation of biological process	9 (12)	5 (10)	0.78
Response to external stimulus	9 (12)	3 (6)	0.36
Morphogenesis	7 (9)	3 (6)	0.73
Organ development	6 (8)	5 (10)	0.75
			
*Molecular Function*			
Protein binding	33 (40)	24 (40)	1
Ion binding	27 (33)	15 (25)	0.35
Nucleic acid binding	18 (22)	12 (20)	0.84
Transferase activity	14 (17)	3 (5)	0.04 (1)^3^
Transcription factor activity	8 (10)	3 (5)	0.36
Receptor activity	7 (9)	11 (18)	0.12
Nucleotide binding	7 (9)	4 (7)	0.76
Hydrolase activity	6 (7)	9 (15)	0.17
Enzyme inhibitor activity	6 (7)	4 (7)	1
Receptor binding	5 (6)	5 (8)	0.74

^1 ^74 genes with Gene Ontology annotation for biological process and 82 for molecular function; ^2 ^50 genes with Gene Ontology annotation for biological process and 60 for molecular function; ^3 ^FDR adjusted p-value.

**Table 4:** Primer sequences and PCR conditions

*Gene*	*Primer sequences*	*T_*a *_(C°)*	*Cycles*
GAPDH	F: 5'-caccaccatggagaaggctgg-3' R: 5'-ccaaagttgtcatggatgacc-3'	65	30
COL3A1	F: 5'-agcctccaactgctccta-3' R: 5'-gtccgggtctacctgatt-3'	56	30
ENO2	F: 5'-gtctgctgctcaaggtcaac-3' R: 5'-tccaggcaagcagaggaatc-3'	54	30
FGF18	F: 5'-cttcctgctgctgtgcttcc-3' R: 5'-cactccttgctggtgccatc-3'	57	30
JUN	F: 5'-gctatctaggtggagttg-3' R: 5'-gcacatgccacttgatac-3'	46	30
LHX2	F: 5'-tgaaggacagcctggtctac-3' R: 5'-gagctgcttcaagtccttgg-3'	56	30
MEIS1	F: 5'-gctgttccagcatctaacac-3' R: 5'-tgttgctgaccgtccattac-3'	50	30
MEIS2	F: 5'-gatcacgccgttatgttgcc-3' R: 5'-gctggagttcgagtgatgag-3'	49	30
PBX3	F: 5'-caggaagcaggacatcgg-3' R: 5'-ttggctctgtaatctgagtgtt-3'	56	30
PDGFRL	F: 5'-gtggagctaccctgcgtatc-3' R: 5'-ctgggagaaggtacaaagagttc-3'	60	35
PRICKLE1	F: 5'-aggtacggtattgccagtctt-3' R: 5'-cgaacactgcaacttcacctc-3'	60	35
SAT	F: 5'-tcactcgccgaggttccttg-3' R: 5'-acagcagcactcctcactcc-3'	53	30
SHB	F: 5'-gtttaatggcaacgagaagcg-3' R: 5'-tcctcacagccacgggatag-3'	60	35
TIMP2	F: 5'-ggaaacgacatttatggcaacc-3' R: 5'-acccagtccatccagaggc-3'	60	30
TLX1	F: 5'-acctcactggcctcaccttc-3' R: 5'-cagaccacggctgcagattc-3'	57	30
TOP1	F: 5'-gaaggaacagctagcagatg-3' R: 5'-agaactctgcctcttgagac-3'	50	30
UACA	F: 5'-cactgaatgacacgttagcca-3' R: 5'-atcctgcacctttctcatgct-3'	60	35

T_a_, annealing temperature.
